# Meteorological for March

**Published:** 1880-04

**Authors:** 


U. S. SIGNAL OFFICE, BALTIMORE, MD., April 1, 1880.

### METEOROLOGICAL SUMMARY FOR THE MONTH OF MARCH, 1880.

| | |
|---|---|
| Mean Monthly Thermometer | 43°.2 |
| " " " | for past nine (9) years, 42°.7 |
| Highest Thermometer | 76° on the 5th |
| Lowest " | 22° on the 25th |
| Monthly Range of Temperature | 54° |
| Mean Daily Range of Temperature | 15° |
| Mean Barometer | 30.096 inches |
| Highest Barometer | 30.515 " on the 12th |
| Lowest " | 29.351 " on the 27th |
| Mean Relative Humidity | 63.6 per cent. |
| Prevailing Direction of Wind | Northwest |
| Highest Velocity of Wind, 28 miles per hour, N. W., on the 5th and 24th. | |
| Total Movement of Wind | 5295 miles |
| Mean Hourly Velocity of Wind | 7.1 " |
| Total No. of Days on which Rain or Snow Fell | 18 |
| Total Rainfall and Melted Snow | 4.82 inches |
| Average Precipitation during March for past 9 years | 3.51 " |

Note.—The Mean Temp. of March, 1880, was only 1°.5 above that of the preceding February, the average difference for 10 years being 5°.7.

ROBERT SEYBOTH,
Observer.

